# Roles for the FCRL6 Immunoreceptor in Tumor Immunology

**DOI:** 10.3389/fimmu.2020.575175

**Published:** 2020-10-14

**Authors:** Randall S. Davis

**Affiliations:** Departments of Medicine, Microbiology, and Biochemistry & Molecular Genetics, The Comprehensive Cancer Center, University of Alabama at Birmingham, Birmingham, AL, United States

**Keywords:** lymphocytes, inhibitory signaling, regulation, tumor immunology, cell-mediated immunity, FCRL family

## Abstract

Members of the Fc receptor-like (*FCRL1–6*) gene family encode transmembrane glycoproteins that are preferentially expressed by B cells and generally repress responses via cytoplasmic tyrosine-based regulation. Given their distribution and function, there is a growing appreciation for their roles in lymphoproliferative disorders and as immunotherapeutic targets. In contrast to FCRL1–5, FCRL6 is distinctly expressed outside the B lineage by cytotoxic T and NK lymphocytes. Its restricted expression by these orchestrators of cell-mediated immunity, along with its inhibitory properties and extracellular interactions with MHCII/HLA-DR, represent a newly appreciated axis with relevance in tolerance and cancer defense. The significance of FCRL6 in this arena has been recently demonstrated by its upregulation in HLA-DR^+^ tumor samples from melanoma, breast, and lung cancer patients who relapsed following PD-1 blockade. These findings imply a potential mechanistic role for FCRL6 in adaptive evasion to immune checkpoint therapy. Here we review these new developments in the FCRL field and identify new evidence for the prognostic significance of FCRL6 in malignancies that collectively indicate its potential as a biomarker and therapeutic target.

## Introduction

The immune system maintains a careful balance of activation vs. inhibition signals to coordinate restraint at homeostasis and promote effector responses when triggered. These cellular mechanisms establish tissue surveillance and stand ready to mount a vigorous immune defense, but must also suppress overzealous responses that could potentially harm the host. The growing significance of inhibitory receptors in immune regulation and human health is underscored by their roles in a variety of disorders including infectious diseases, autoimmunity, and cancer.

The discovery that malignancies have evolved mechanisms that exploit inhibitory receptors to circumvent elimination by immune cells is fundamentally impacting our understanding of tumor immunology and revolutionizing cancer therapy. Antibody (Ab)-mediated targeting of the PD-1/PD-L1 and CTLA-4/B7 immune checkpoint inhibitor (ICI) axes enables disruption of receptor-ligand interactions that shield tumors from infiltrating cytotoxic lymphocytes (1, 2). This selective approach has reinvigorated the field of tumor immunology and ignited extraordinary potential for new diagnostic, prognostic, and therapeutic strategies that deliver more targeted and effective patient care. As of 2018, it is estimated that ~44% of cancer patients are eligible for ICI therapy (3). However, as treatment expands, many patients who enjoyed durable responses will relapse as the tumor adapts and becomes resistant to recognition and rejection by tumor infiltrating lymphocytes (TILs) (4, 5). Unfortunately, the mechanisms responsible for ICI resistance remain incompletely defined. This issue is becoming a growing barrier for cancer patients who have limited therapeutic options and require alternative strategies to overcome the tumor's adaptive resistance.

**Figure 1:**
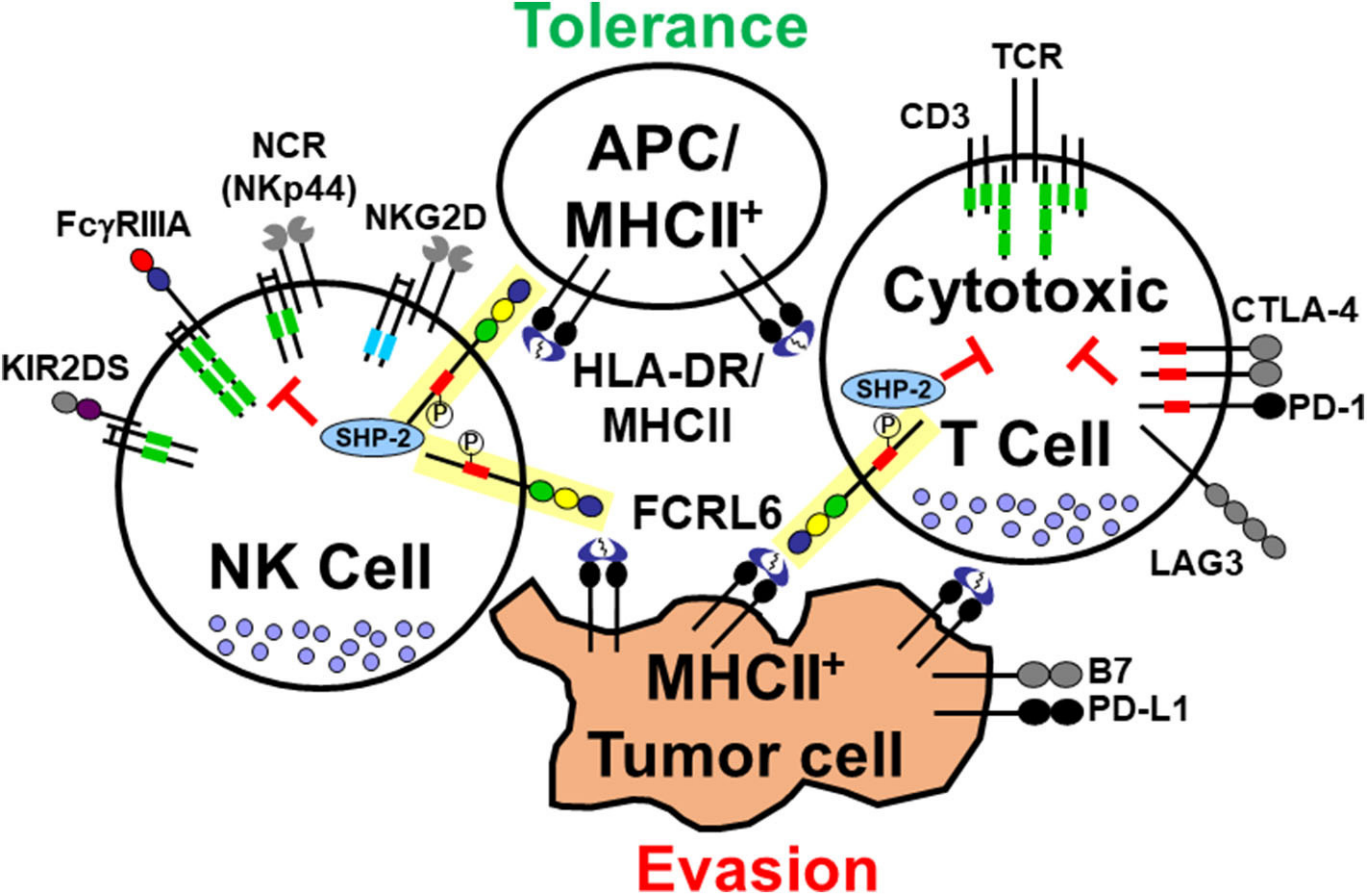
Interactions of the cytotoxic lymphocyte-expressed FCRL6 immunoreceptor with MHCII-expressing cells. The schematic representation shows the FCRL6 receptor (yellow box), which is composed of three extracellular Ig-like domains, an uncharged transmembrane region, and a cytoplasmic tail with two tyrosines, including a consensus ITIM (red rectangle) that recruits the SHP-2 tyrosine phosphatase. FCRL6 is expressed by cytotoxic T and NK cells and has been found to interact with MHCII/HLA-DR. Other pertinent adaptor-associated activating (ITAM-bearing, green rectangles and DAP-10, blue rectangles) and immune checkpoint inhibitory (ICI) molecule pairs (CTLA-4/B7, PD-1/PD-L1, LAG3/MHCII, and NKp44/MHCII) highlight the regulatory balance of immune tolerance and tumor evasion between cytotoxic lymphocytes and APCs or tumor cells. Note NKp44 also has an inhibitory splice isoform (6).

Here we review recent developments related to members of the Fc receptor-like (FCRL1–6) immunoregulatory family with a specific focus on the FCRL6 molecule in cell-mediated immunity and its newly appreciated roles in tumor immunology. Its restricted expression by cytotoxic NK and T cells, cytoplasmic tyrosine-based inhibitory properties, and extracellular interactions with MHCII/HLA-DR introduce a new axis with relevance in tolerance and cancer defense (Figure 1). Its importance was recently demonstrated in studies that identified its upregulation in HLA-DR^+^ tumor samples from melanoma, breast, and lung cancer patients who had relapsed following PD-1 blockade (7). These findings imply a potential mechanistic role for FCRL6 in adaptive evasion to ICI therapy. By investigating its expression among The Cancer Genome Atlas (TCGA) tumor samples, we identify new evidence for the prognostic significance of FCRL6 in melanoma, breast, and lung cancer that collectively indicate its potential as a biomarker and therapeutic target.

## FC Receptor-Like Molecules (FCRL) in B Cell Regulation

An extended family of *FCRL1–6* genes in humans and mice encode type I transmembrane (TM) glycoproteins with cytoplasmic immunoreceptor tyrosine-based activation (ITAM)-like or inhibitory (ITIM) motifs [reviewed in (8, 9)]. FCRL1–5 are preferentially expressed by B lineage cells and modulate B cell antigen-receptor (BCR)-mediated signaling (10). Notably, FCRL3 is also detected outside the B lineage on subsets of T and NK cells (11–13). While FCRL1 is a pan B cell marker with ITAM-like motifs that promotes BCR activation in humans and mice [unpublished studies and (14–16)], signaling studies demonstrate that FCRL2–5 generally exert inhibitory function. Consistent with their possession of cytoplasmic ITIM sequences, following BCR cross-linking, FCRL2–5 are tyrosine phosphorylated (pY) and can repress global pY, Ca^2+^ flux, and MAP kinase activation via recruitment of the SHP-1 and/or SHP-2 phosphatases (17–21). In mice (m), mFCRL5 has similar inhibitory properties (22). However, the presence of both ITAM and ITIM in the FCRL2–5 representatives implies more complex signaling than the classical FCRs that are either activating or inhibitory (23). Accordingly, mFCRL5 has the ability to modulate BCR signaling in a binary fashion. However, its functional properties among B cell subsets vary according to the differential recruitment of the Lyn Src-family kinase (SFK) to an ITAM-like sequence and SHP-1 to an ITIM (21). The capacity for composite activating and inhibitory signaling is also present in human FCRL3 and FCRL4. These proteins possess dual regulatory properties that appear to differ according to the innate (Toll-like receptor/TLR) or adaptive (BCR) nature of B cell stimulation (24, 25). Molecular dissection of the FCRL4 cytoplasmic tail has demonstrated that its function is altered by the recruitment of at least two different SFKs. FCRL4 wields suppressive activity in B cells co-expressing FGR, but promotes activation in B cells co-expressing HCK p59 (26). Thus, as opposed to the classical FCR for IgG and IgE, these findings highlight that many FCRLs possess multifaceted regulatory potential.

Beyond their intracellular signaling capability, ligands have been identified for several FCRLs. The potential for Ig binding was initially detected for FCRL4 and FCRL5, but was confirmed in studies by Wilson et al. who found these receptors could interact with IgA and IgG (27, 28). However, unlike the classical IgG and IgE FCRs, IgG binding to FCRL5 is glycosylation dependent and requires both the Fab and Fc portions (29). Recent work has also identified secretory IgA as a ligand for FCRL3 and this relationship could impact inhibition in T regulatory cells (30). How these receptor-ligand interactions influence the systemic and cellular function of the lymphocytes that express them or contribute to disease pathogenesis remains ripe for future investigation.

## Relevance of FCRL1-5 in B Cell Lymphoproliferative Disorders

The preferential expression of FCRL1–5 by B cells has made these molecules relevant clinical candidates in lymphoproliferative disorders such as leukemias and lymphomas. In fact their initial identification by the Dalla-Favera group as immunoglobulin superfamily receptor translocation-associated (IRTA) genes, resulted from the characterization of a t(1;14)(q21;q32) translocation breakpoint in a multiple myeloma cell line (28). These early studies also demonstrated dysregulated *FCRL5/IRTA2* transcript expression in follicular lymphoma and myeloma cell lines with 1q21 abnormalities (31). Variable upregulation of *FCRL1–5* in B cell malignancies was further revealed through the Lymphochip-based microarray analyses led by the Staudt group (32), but have now been expanded in ever-growing numbers of high-throughput RNA-seq studies. Following the development of monoclonal Abs (mAbs), several groups validated FCRL surface protein expression by malignant B cell lines as well as B cell chronic lymphocytic leukemia (CLL) cells, the most common leukemia in Western countries (11, 33, 34). An analysis of FCRL1–5 in a cohort of CLL patients well-characterized for standard prognostic factors, identified FCRL2 as a marker for a subgroup of CLL patients with a more indolent disease course as reflected by favorable progression free survival and overall survival (34, 35). By flow cytometry, FCRL2 was able to segregate CLL samples according to the mutation status of the *IGHV* gene expressed by the leukemic clone. Its upregulation by mutated *IGHV* CLL samples introduced a novel surface marker of this favorable disease subtype. This pattern of FCRL2 expression was in contrast to CD38, ZAP-70, and CD49d, which are chiefly upregulated in patients with unmutated CLL who experience a more aggressive disease course (10, 36, 37). The inhibitory function evident for FCRL2 in healthy B cells (19) implies that its upregulation by indolent *IGHV* mutated CLL might also contribute to biological suppression in this favorable CLL subtype. Thus, the expression of these regulatory proteins may not only have prognostic significance, but could also have physiological impact on the clinical disease course and pathogenesis of certain lymphoproliferative disorders.

The targetability of FCRL members in B cell malignancies is an area of active investigation and FCRL5 has become a promising therapeutic candidate. Analysis of blood and tissue samples by the Pastan laboratory identified elevated levels of soluble and surface bound FCRL5 in multiple myeloma, CLL, and mantle cell lymphoma patient samples (33). Studies targeting FCRL5 in myeloma have been conducted by investigators at Genentech (38–40). Two approaches have been pursued including a mAb drug conjugate and a T cell-dependent bispecific mAb. Pre-clinical xenograft models employing multiple myeloma samples indicated efficacy for a humanized IgG1 isotype FCRL5 mAb conjugated to monomethyl auristatin E (MMAE) (38). This microtubule inhibitor becomes active when surface receptors bound by the mAb-conjugate are internalized in target cells. A Phase 1 study of this anti-FCRL5-MMAE drug compound (DFRF4539A) in relapsed and refractory multiple myeloma patients (*n* = 39) showed tolerability as a single agent, but demonstrated limited activity and may not be a successful strategy for myeloma (39). The authors speculated that the low response rates in this study could be due to the unknown threshold for Ab-dependent cytotoxicity activity, shedding of the FCRL5 target, limited internalization, and a less effective role of MMAE in cells with a low proliferative index. However, preclinical studies with a bispecific FCRL5/CD3 mAb has shown encouraging activity against patient myeloma cells, can deplete B cells and bone marrow plasma cells in cynomolgus monkeys, and exhibits enhanced activity when combined with PD-L1 blockade (40). These studies indicate that immunotherapeutic targeting of FCRL5 and other FCRL family members expressed by B cells may have utility in a variety of lymphoproliferative disorders.

## Human FCRL6 Is an Immunoregulatory Protein Restricted to Cytotoxic T and NK Cells

Notably, the first member of the FCRL family identified was a rat FCRL6 ortholog, termed gp42, that was discovered in a search for markers of lymphokine activated killer (LAK) cells (41). Following the discovery of human FCRL1–5, we and others identified human and mouse *FCRL6*/*Fcrl6* counterparts, both of which encode type I TM glycoproteins (42–44). However, the structure and distribution of these FCRL6 molecules among lymphocytes has marked interspecies differences (43). Recent studies with receptor-specific mAbs identified the expression of mFCRL6, which has two Ig like extracellular domains and a short cytoplasmic tail lacking a consensus ITIM or ITAM, by subsets of progenitor B cells in the fetal liver and bone marrow (45). An analysis of pro B cell subsets purified according to the presence or absence of FCRL6 expression, revealed that FCRL6^+^ progenitors have a distinct transcript signature, constrained diversity of their *IGHV* repertoires, and hydrophobic and charged CDR-H3 characteristics akin to innate-like B-1 cells that produce natural Abs (45). However, the regulatory role of mFCRL6 among these pro B cells is not yet defined. Despite syntenic genomic positions, the disparate structure and expression pattern of this family member presents some barriers for *in vivo* translational understanding of its human relative. In contrast, human FCRL6, which has three extracellular Ig-like domains, an uncharged TM region, and a cytoplasmic tail featuring a consensus ITIM, is restricted to cytotoxic T and NK cells (44, 46).

The development of receptor-specific mAbs facilitated examination of its ontogeny and distribution in human tissues. FCRL6 is present on mature NK and T lymphocytes from adult spleen and blood, but not by these cells from primary developmental sites such as the fetal liver, bone marrow, or thymus (45). Inflammatory tonsillar tissue also lacks significant numbers of FCRL6^+^ cells. Within the blood, FCRL6 marks more terminally differentiated cytotoxic CD16^+^CD56^dim^ NK cells, a finding that correlates with cytoplasmic perforin and expression of the keratin sulfate-related lactosamine carbohydrate epitope, PEN5 (47). The possibility that FCRL6 expression increases as a function of ontogeny was also confirmed by comparing CD16^+^ NK cells isolated from cord and adult blood samples. FCRL6 is expressed at significantly higher levels among circulating adult CD16^+^ cells suggesting that it emerges later in ontogeny than CD16 and segregates NK cells that are more mature (45). Within the T cell compartment, FCRL6 is present on innate-like γδ T cells, but is not biased among Vδ1 or Vδ2 subsets. However, its expression by γδ T cells similarly correlates with perforin and is relatively more abundant on CD16^hi^ Vδ2 cells that have greater cytotoxic propensity (48). Among CD8^+^ T cells, FCRL6 is primarily restricted to perforin-expressing effector (CD45RA^+^CCR7^−^ or CD28^−^2B4^+^) and effector memory (CD45RA^−^CCR7^−^ or CD28^+^2B4^+^) subpopulations, rather than central memory or naïve cells. Interestingly, a small (~2%), but consistent population of FCRL6^+^CD4^+^ T cells that co-express perforin, CD57, and NKG2D, but lacks CCR7 is present in the blood of some donors (46). Such rare CD4^+^ T cells possess cytolytic function (49). These data indicate that FCRL6 distinctly marks mature NK and T cell subpopulations with cytotoxic potential.

## FCRL6 Recruits SHP-2 to an ITIM and Is an MHCII/HLA-DR Ligand

The presence of two tyrosines in the FCRL6 cytoplasmic tail suggests that, like other FCRLs, it harbors regulatory function. One of these tyrosines (Y371) is positioned among amino acids that conform to a consensus ITIM, but the sequence surrounding both tyrosines (Y356, Y371) could represent a non-canonical ITAM. GST pull down assays of Y356F and Y371F mutants performed with Jurkat lysates uncovered recruitment of the SHIP1 inositol phosphatase as well as the GRB2 adapter protein to the Y356 residue and the SHP1/SHP2 tyrosine phosphatases to the Y371 site (50). Immunoprecipitation of FCRL6 from pervanadate treated NK or T cells validated its capacity for pY and interactions with multiple pY proteins [unpublished data and (46)], including SHP-2 to the Y371 residue (44). These findings imply an inhibitory role for FCRL6 in cytotoxic lymphocytes. However, an important question for understanding the fundamental biology of FCRL6 is the nature of its extracellular partner(s).

In flow cytometry-based studies, we were unable to detect Ig binding to FCRL6 by surface staining (46). To search for FCRL6 binding partners, we engineered a cell-based GFP reporter system that expressed a chimeric receptor comprised of the FCRL6 ectodomain in frame with the cytoplasmic tail of mouse CD3ζ (51). In co-culture assays with various cell types, the FCRL6-CD3ζ reporter line was activated by antigen presenting cells (APCs) including B lymphocytes and dendritic cells. This work led to identifying MHCII/HLA-DR as an FCRL6 ligand. Furthermore, interactions between FCRL6 and HLA-DR appeared to differ according to the nature of the β chain component of the heterodimer. This observation suggests that binding affinities between FCRL6 and MHCII may differ according to HLA-DR haplotype. Within the context of this finding FCRL6 is not unique. Several other surface immunoreceptors have also been found to bind MHCII (see Figure 1). Intriguingly, the LAG-3 immunoreceptor, a CD4 relative also expressed by T cells, is upregulated by exhausted cells in chronic immune conditions including malignancies, and exhibits inhibitory effects through interactions with MHCII/HLA-class II (52, 53). Notably, LAG3 has become an attractive immunotherapeutic target and at least three LAG3-specific ICI mAbs are in development (54). However, recent work has identified additional non-MHCII ligands for LAG3 (55). Furthermore, a third MHCII receptor expressed by NK cells was recently identified. The natural cytotoxicity receptor NKp44, which has different isoforms as well as expression outside the NK lineage (6), was found to bind subsets of MHCII/HLA-DP molecules (56). These findings collectively indicate the existence of multiple immunoreceptors that may serve to modulate relationships between cytotoxic lymphocytes and MHCII-expressing cells in different settings [recently reviewed by (57)].

## FCRL6-MHCII Interactions Repress Effector Functions by Cytotoxic Lymphocytes

Efforts to investigate the functional properties of FCRL6 were initially unrevealing. While the genetic regulation of *FCRL6* has not yet been explored in detail, modulation experiments showed that the receptor is down-regulated from the NK cell surface when exposed to activating cytokines such as IL-2, IL-12, or IL-15 and by CD8^+^ T cells upon anti-CD3 activation [(44) and our unpublished data]. This analysis suggests that FCRL6 is sensitive to cellular activation. Beyond its induction, re-directed killing assays with FCRL6-expressing NK-92 transfectants or freshly isolated NK or CD8^+^ T cells were used to target receptor-specific mAb-coated P815 cells for cytolysis. Work by our group and the Colonna laboratory found no effect for FCRL6 on NK cell degranulation (surface LAMP1 detection), cytoplasmic IFNγ production, or the anterograde cytolysis efficiency of ^51^Cr-labeled P815 target cells (44). Furthermore, FCRL6 did not influence activation receptor (CD16)-mediated killing. A potential regulatory role for FCRL6 on cytokine production was also underwhelming. Cultured CD56^+^ NK cells cross-linked with FCRL6 mAbs demonstrated only slight increases in IFNγ and TNFα production when IL-2 was present, but no impact was found for IL-4, IL-5, or IL-10 (44). Furthermore, no differences in cytokine production were observed in similar studies with CD8^+^ T cells +/– anti-CD3 or IL-2. Thus, FCRL6 does not appear markedly influence cytokine generation by these cytotoxic lymphocytes *in vitro*.

In recent studies that identified the upregulation of FCRL6 and LAG3 in the microenvironment of HLA-DR^+^ solid tumors [detailed below and (7)], we revisited FCRL6 function with respect to its MHCII ligand. The regulation of NK cell cytotoxicity by MHC class I molecules has been well-characterized, and serves as the basis for the “missing-self” hypothesis (58), but evidence also exists that MHC class II expression can protect target cells from NK cell-mediated killing. Early work demonstrated that enforced expression of HLA-DR by K562 cells, a classic human erythroleukemic MHCII–negative target cell line, could inhibit lysis by freshly isolated human NK cells (59). Furthermore, transplantation of HLA-DR^+^ K562 cells into NOD/SCID mice provided protection of tumors from elimination by adoptively transferred human blood NK cells (60). We thus generated NK-92 FCRL6 transductants and used them for cytotoxicity assays by employing HLA-DR^+^ K562 target cells. These experiments demonstrated that HLA-DR expression by K562 cells inhibited the cytotoxicity of FCRL6-expressing NK cells (7). Additional support for this inhibitory axis was found for CD8^+^ T cell responses. By employing an FCRL6 mAb that disrupts HLA-DR binding to FCRL6-CD3ζ reporter cells and in cell staining assays (51), we investigated the effect of FCRL6 blockade during pathogen-specific peptide stimulation *in vitro*. The addition of FCRL6 or PD-L1 blocking mAbs to healthy donor mononuclear cells co-cultured in MHC class I–restricted peptides from CMV, EBV, and influenza virus epitopes, resulted in enhanced frequencies of IFNγ and TNFα cytokines upon restimulation (7). These studies indicate that FCRL6 is a potentially novel ICI target capable of suppressing effector cell activity following engagement with HLA-class II molecules.

## FCRL6^+^ NK and T Lymphocytes Expand in Chronic Immune Disorders

The significance of FCRL6 in immune-related disorders was first shown by Wilson et al. who found significantly expanded FCRL6^+^ effector and effector memory CD8^+^ T cell frequencies in HIV-1 infected individuals (44). This expression pattern did not seem to correlate with viral titers or CD4^+^ counts. An increase in circulating FCRL6^+^CD4^+^ cells, which are typically rare among healthy individuals, was also detected in HIV^+^ donor samples. Initial evidence for a role in tumor immunology came from studies in CLL. Unlike the other FCRL molecules that are expressed by CLL B cells (34), FCRL6 expression by the B cell clone was undetectable. Instead, increased frequencies of FCRL6^+^ NK and T lymphocytes were evident in the circulation of CLL patients (46). While blood CD8^+^ T cells are generally expanded in CLL, T cells derived from these patients are also known to have abnormal function (61, 62). An analysis of CLL donors from our cohort demonstrated that, in addition to a global increase in CD8 T cells, the frequencies of effector and effector memory cell populations were elevated compared to healthy control samples (46). The frequency of FCRL6^+^ cells among these CD8^+^ subsets was also greater in CLL patients, as were FCRL6-expressing NK cells and cytotoxic CD4^+^ cells. These findings suggest that the expansion of FCRL6^+^ cytotoxic T and NK cells in different disease states could reflect the influence of chronic immune activation on the terminal differentiation of effector cells and contribute to their dysfunction. Furthermore, the increased numbers of cytotoxic cells marked by FCRL6 could reflect a blunted capacity for clearance of the inciting disease process by taking advantage of its potential inhibitory properties. This could infer a role for cytokines or some other systemic influence on the expansion of FCRL6^+^ cells, but the mechanistic basis for this remains undefined.

## FCRL6 Expression Is Upregulated in Solid Tumors Expressing HLA Class II

Evidence that an inhibitory receptor restricted to cytotoxic lymphocytes interacts with MHCII/HLA-DR suggests that FCRL6 might have roles in tolerance through its interactions with APCs as well as other non-traditional MHCII-expressing cells including malignancies (see Figure 1). The endogenous expression of MHCII/HLA-DR molecules by tumor cells has been observed in many cancers including 40–50% of melanoma cases (63, 64). Importantly, MHCII expression correlates with favorable clinical responses to anti-PD-1 ICI in melanoma, classical Hodgkin's disease, breast cancer, and ovarian cancer (64–67). In breast cancer, PD-1 inhibition is more efficacious in the triple negative subtype (TNBC) (68), but responses vary by PD-1/PD- L1 expression and TIL frequencies (69). Accordingly, MHCII is expressed by ~30% of TNBC cases (70) and portends increased therapeutic responses and TIL recruitment (71, 72). Despite the clinical favorability of MHCII^+^ status, chronic ICI therapy typically leads to tumor resistance by adaptation and the delivery of immunosuppressive signals through alternative checkpoint pathways (4).

To investigate these evasion mechanisms, in collaboration with Johnson and Balko, we recently performed transcriptome profiling and tissue staining of patients who developed resistance to PD-1 immunotherapy in melanoma, non-small cell lung cancer (NSCLC), and TNBC (18). This study included melanoma and NSCLC samples (*n* = 58) before and after targeted PD-1 ICI. MHCII^+^ tumors, confirmed by dual RNA-*in situ* and immunohistochemistry analysis, demonstrated an adaptive immune signature including the upregulation of genes encoding *CD4, CD8a*, and ICI receptors. Chiefly among these was LAG3, which was exclusively expressed by infiltrating T cells with a bias toward CD8^+^ rather than CD4^+^ cells. A comparison of melanoma samples derived from patients pre and post anti-PD-1 treatment revealed increased frequencies of LAG3^+^ TILs as a function of developing adaptive resistance in paired specimens. High levels of LAG3^+^ TILs were also found in MHCII^+^ TNBC (*n* = 112) and were associated with CD4^+^ infiltrates. Given the known interaction between LAG3 and MHCII (52, 53), these findings implied that tumors may exploit MHCII expression to blunt eradication by TILs. This hypothesis was further supported by studies in an *in vivo* MMTV-neu breast cancer mouse model. Enforced MHCII expression generally promoted tumor rejection and CD4 recruitment, but in mice that formed refractory tumors, there was evidence of adaptive resistance including the upregulation of chemokines that foster T cell recruitment as well as the *Pdcd1* (Pd-1) and *Lag3* inhibitory receptor genes. Importantly, anti-tumor immunity was enhanced by treatment with a combination of anti-PD-1 and LAG3 ICI.

With clinical and mechanistic evidence that MHCII^+^ tumors may actively suppress effector cell cytotoxicity, we turned to the possibility that FCRL6 may operate similarly in this process. As detailed above, FCRL6 suppressed NK cell cytotoxicity of HLA-DR expressing target cells and enhanced effector T cell cytokine production following Ab-mediated blockade. Like LAG3, *FCRL6* was more highly expressed by MHCII^+^ melanomas and NSCLCs. While a similar trend was evident for *FCRL3*, it did not reach significance. A linear relationship was also evident for *LAG3* and *FCRL6* with the degree of HLA-DR^+^ tumor cells. Similarly, both *FCRL6* transcript and protein expression was elevated in melanoma samples from patients who experienced relapse after progression on anti-PD-1 ICI therapy. Consequently, FCRL6^+^ infiltrates also correlated with LAG3 and HLA-DR status in TNBC. Staining of TNBC specimens showed that TIL co-expression of FCRL6 and LAG3 was strongly correlated with elevated tumor-specific HLA-DR expression. Finally, in these breast tumors, an inverse correlation was found for FCRL6 and LAG3 reactivity with the fraction of granzyme B^+^ cytotoxic CD8^+^ cells present. This disclosed a potential suppressive role for these ICI receptors in the tumor microenvironment. These findings collectively implicate a novel inhibitory role for FCRL6 in cell-mediated responses to MHCII^+^ tumors and its potential as a new ICI target that influences adaptive resistance mechanisms to anti-PD-1 therapy.

## FCRL6 Upregulation by Malignancies has Prognostic Significance

**Figure 2:**
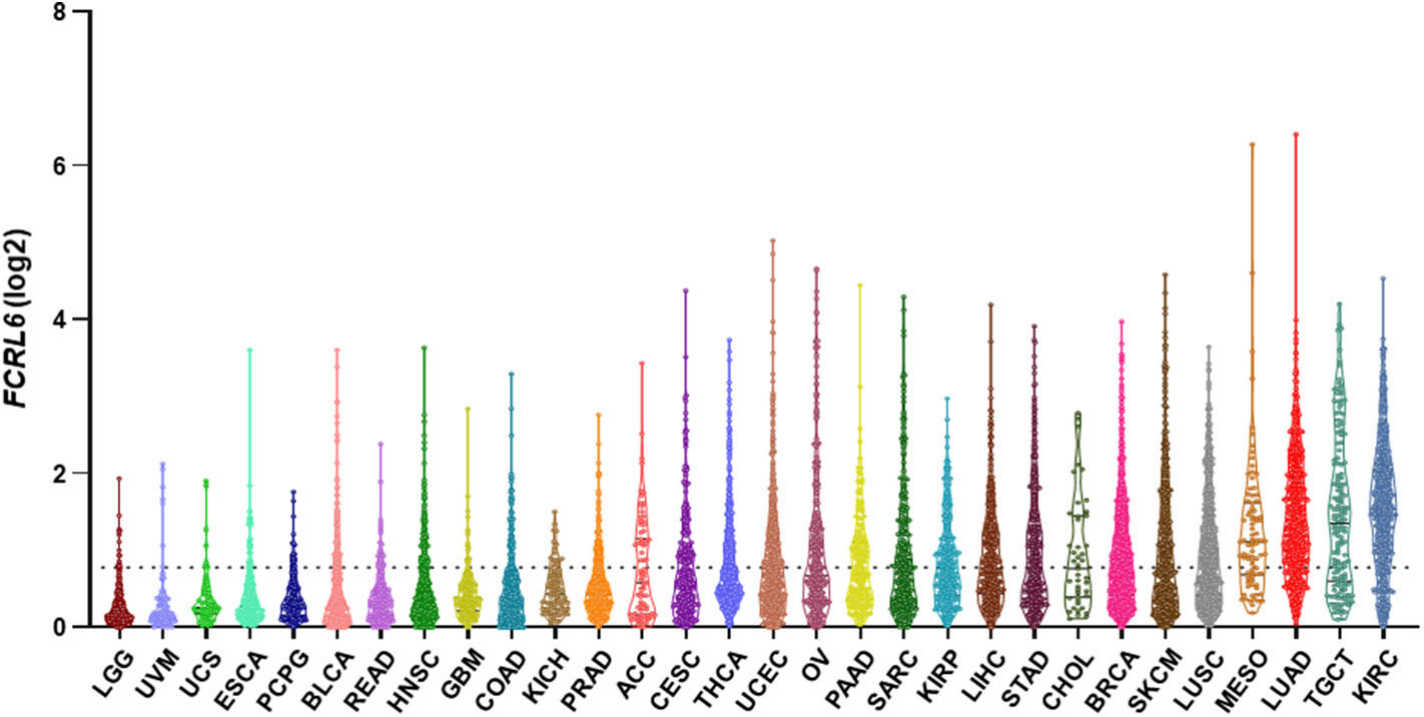
*FCRL6* expression among various cancers. A violin plot demonstrating the expression (log2) of *FCRL6* (ENSG00000181036) in non-hematopoietic cancers (*n* = 30) from The Cancer Genome Atlas (TCGA) (73). LGG, lower grade glioma (*n* = 529); UVM, uveal melanoma (*n* = 80); UCS, uterine carcinosarcoma (*n* = 56); ESCA, esophageal carcinoma (*n* = 173); PCPG, pheochromocytoma and paraganglioma (*n* = 186); BLCA, bladder carcinoma (*n* = 427); READ, rectum adenocarcinoma (*n* = 177); HNSC, head and neck squamous cell carcinoma (*n* = 546); GBM, glioblastoma (*n* = 174); COAD, colon adenocarcinoma (*n* = 499); KICH, kidney chromophobe (*n* = 89); PRAD, prostate adenocarcinoma (*n* = 548); ACC, adrenocortical carcinoma (*n* = 79); CESC, cervical squamous carcinoma (*n* = 309); THCA, thyroid carcinoma (*n* = 568); UCEC, uterine corpus endometrial carcinoma (*n* = 579); OV, ovarian serous cystadenocarcinoma (*n* = 379); PAAD, pancreatic adenocarcinoma (*n* = 182); SARC, sarcoma (*n* = 265); KIRP, kidney renal papillary cell carcinoma (*n* = 320); LIHC, liver hepatocellular carcinoma (*n* = 424); STAD, stomach adenocarcinoma (*n* = 407); CHOL, cholangiocarcinoma (*n* = 45); BRCA, breast carcinoma (*n* = 1,205); SKCM, skin cutaneous melanoma (*n* = 472); LUSC, lung squamous cell carcinoma (*n* = 551); MESO, mesothelioma (*n* = 86); LUAD, lung adenocarcinoma (*n* = 573); TGCT, testicular germ cell tumor (*n* = 156); and KIRC, kidney renal clear cell carcinoma (*n* = 599). The median and quartiles are demarcated (black lines) for samples in the plot of each cancer subtype. RPKM transcript data were downloaded from the R2: Genomics Analysis and Visualization Platform (http://r2.amc.nl), plotted using Prism software, and ordered by median values of expression. The dotted black line indicates the mean expression value (*FCRL6* log2 = 0.78) of the 30 cancer subtypes. Note that THCA and cancers to the right on the plot exceed the mean.

**Figure 3:**
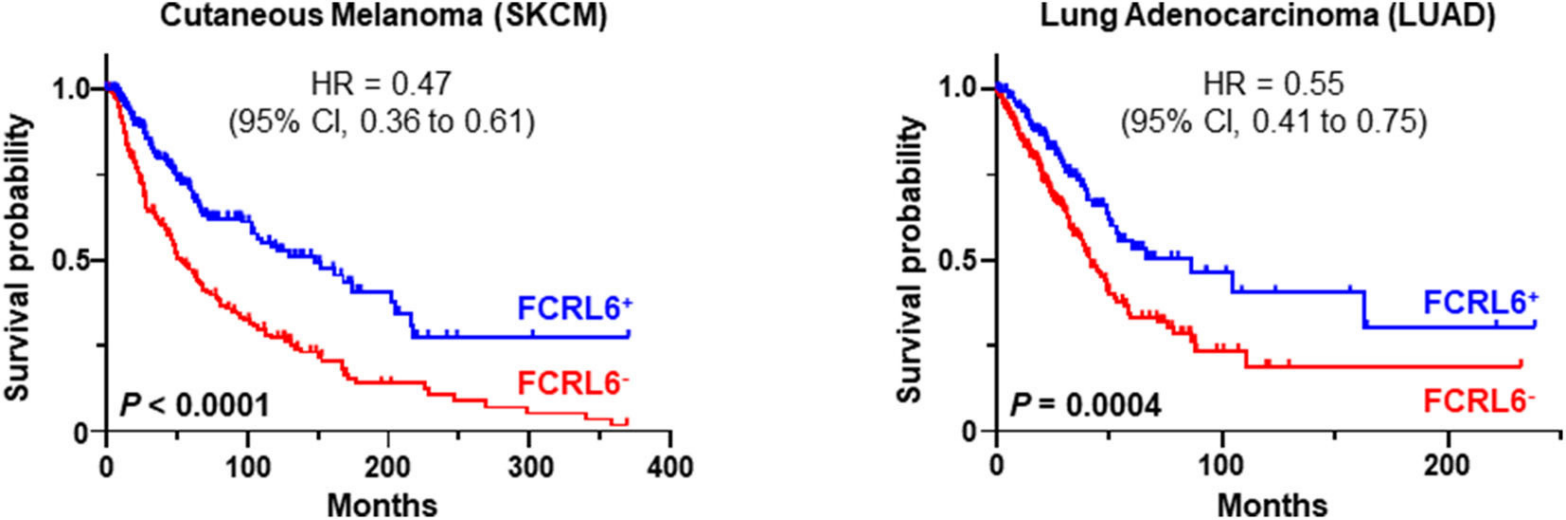
*FCRL6* overexpression predicts favorable overall survival (OS) in cutaneous melanoma and lung adenocarcinoma. Kaplan-Meier plots demonstrating the relationship between *FCRL6* gene expression and patient clinical outcomes by OS. TCGA (reads per kilobase million—RPKM) transcript data for SKCM (*n* = 458) and LUAD (*n* = 497) were downloaded from the publicly available cBioPortal (https://www.cbioportal.org/) database (74) for analysis with the R2: Genomics Analysis and Visualization Platform (http://r2.amc.nl). Optimal threshold cut-off values for determining high or low *FCRL6* expression as a continuous variable were compared using Cox Regression analysis and the R2 Genomics platform. Comparisons of OS curves and *P*-values were made using the Log-rank test. Hazard ratio (HR) and 95% confidence interval (CI) for comparisons between the groups are indicated. Kaplan-Meier plots were generated using Prism software.

Given its discrete expression by cytotoxic T and NK lymphocytes and newfound role in tumor immunity, we explored the possibility that detection of *FCRL6* in the tumor microenvironment could have prognostic clinical significance. To pursue this hypothesis, we analyzed *FCRL6* transcript expression from RNA-sequencing data performed on 30 non-hematopoietic cancer types (10,683 samples) from TCGA (73) (Figure 2). *FCRL6* expression (log2) was heterogeneous among these cancer types but, in accord with our recent findings (7), lung adenocarcinomas (LUAD), cutaneous melanomas (SKCM), and breast carcinomas (BRCA) were among the tumors with higher median expression levels. We next assessed survival in these cancer types according to *FCRL6* expression. By employing Cox Regression analysis along with the R2: Genomics Analysis and Visualization Platform (http://r2.amc.nl), we defined optimized cut-off values to segregate samples according to *FCRL6* expression. Kaplan-Meier plots demonstrated that higher *FCRL6* expression predicted increased overall survival (OS) in SKMM (n = 458) and LUAD (*n* = 497) TCGA samples (Figure 3). In SKMM, median OS was 2.7-fold higher for patients with elevated *FCRL6* expression. Patients with *FCRL6*-positive tumors had a median OS of 148.2 months vs. 54.4 months for samples with low *FCRL6* transcripts (HR = 0.47, CI = 0.36–0.61, *P* < 0.0001). In LUAD, elevated *FCRL6* predicted a 2.1-fold higher median OS. *FCRL6*^+^ tumors exhibited a median OS of 86.0 months vs. 41.7 months for *FCRL6*^−^ samples (HR = 0.55, CI = 0.41–0.75, *P* = 0.0004). Consistent with the known responsiveness of these tumors to ICI therapy and the TIL concentrations in the tumor microenvironment (75–82), these findings indicate that elevated *FCRL6* expression confers a generally favorable prognosis for the OS of patients with these tumors.

**Figure 4:**
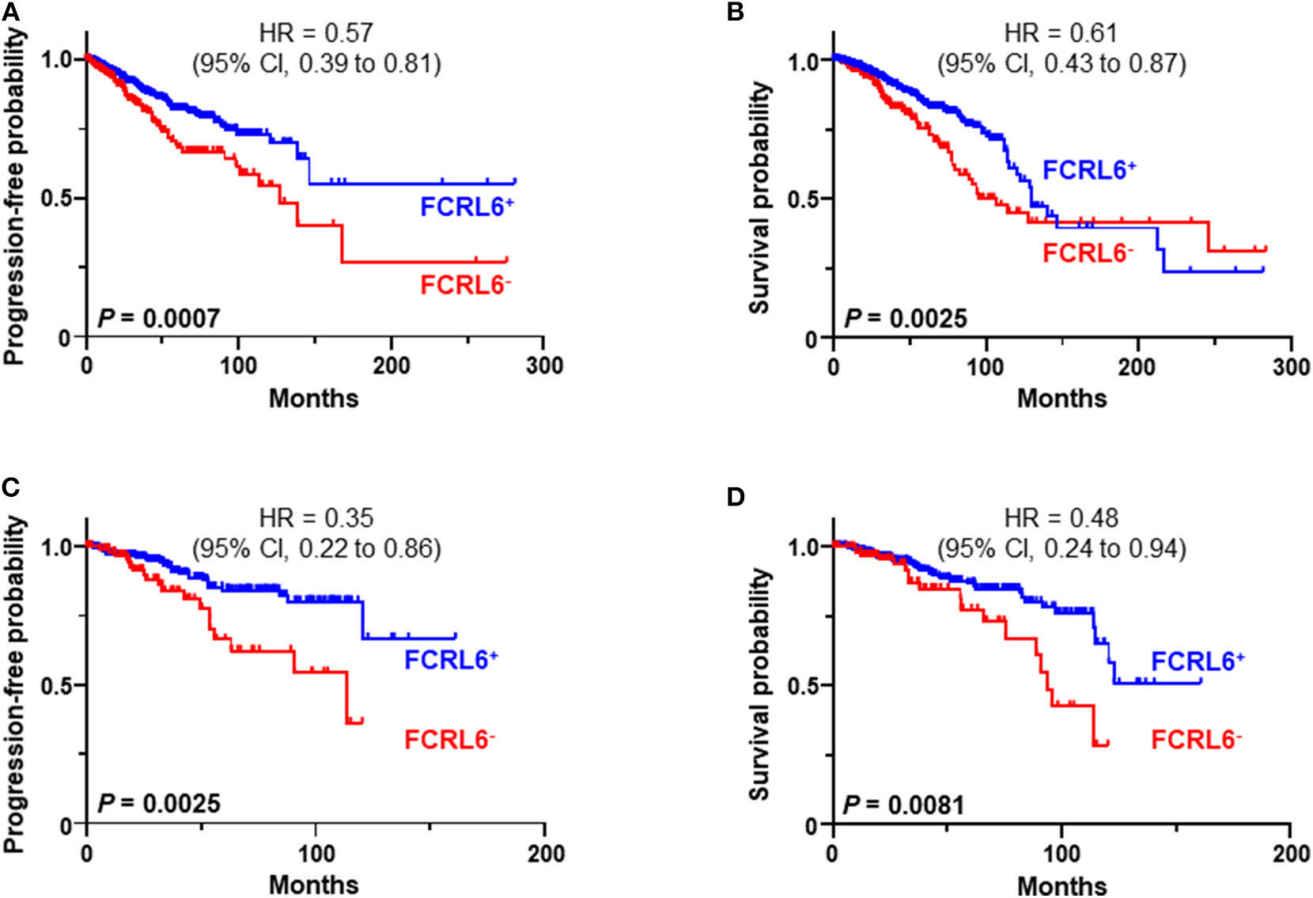
*FCRL6* overexpression predicts favorable progression free (PFS) and overall survival (OS) in breast carcinoma. Kaplan-Meier plots demonstrating the relationship between *FCRL6* gene expression and patient clinical outcomes by PFS and OS for **(A,B)** all breast carcinomas (BRCA, *n* = 1,067) and **(C,D)** Her2 negative samples (*n* = 550). TCGA (reads per kilobase million—RPKM) transcript data for BRCA were downloaded from the publically available cBioPortal (https://www.cbioportal.org/) database (74) for analysis with the R2: Genomics Analysis and Visualization Platform (http://r2.amc.nl). Optimal threshold cut-off values for determining high or low *FCRL6* expression as a continuous variable were compared using Cox Regression analysis and the R2 Genomics platform. Comparisons of PFS and OS curves and *P*-values were made using the Log-rank test. HR and 95% CI for comparisons between the groups are indicated. Kaplan-Meier plots were generated using Prism software.

**Figure 5:**
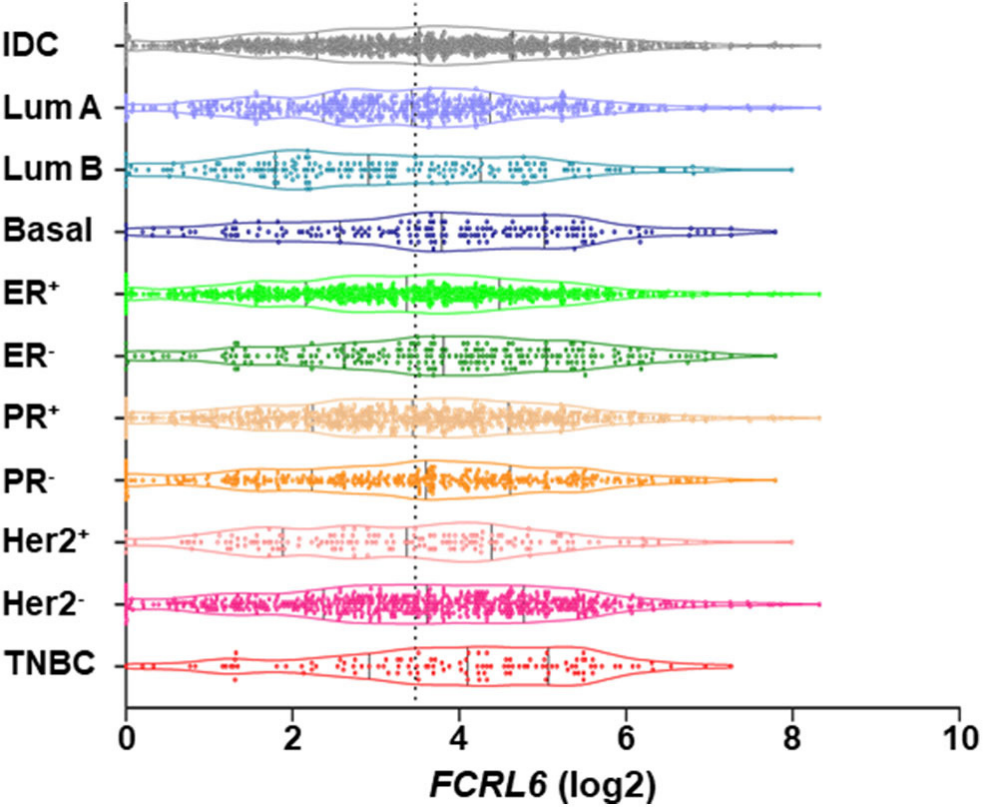
Among breast cancer subtypes *FCRL6* transcript expression is highest in TNBC. A violin plot demonstrating the mean expression (log2) of *FCRL6* (ENSG00000181036) among TCGA BRCA tumor subtypes downloaded from the publicly available cBioPortal (https://www.cbioportal.org/) database (74). Intraductal carcinoma (IDC, *n* = 976), Luminal A (Lum A, *n* = 499), Luminal B (Lum B, *n* = 197), Basal (*n* = 171), estrogen receptor positive (ER^+^, *n* = 795), ER negative (ER^−^, *n* = 237), progesterone receptor positive (PR^+^, *n* = 687), PR negative (PR^−^, *n* = 342), Her2 receptor positive (Her2^+^, *n* = 160) Her2 receptor negative (Her2^−^, *n* = 557), and triple negative breast cancer (TNBC, *n* = 115). The median and quartiles are demarcated (black lines) for samples in the plot of each subtype. Mean (*x*) *FCRL6* expression ± SEM: TNBC (3.905 ± 0.1455), ER^−^ (3.76 ± 0.1104), Basal (3.733 ± 0.1296), Her2^−^ (3.543 ± 0.07162), IDC (3.466 ± 0.05328), PR^−^ (3.449 ± 0.09192), PR^+^ 3.396 ± 0.06334), Lum A (3.384 ± 0.07137), ER^+^ (3.316 ± 0.05859), Her2^+^ (3.263 ± 0.1262), and Lum B (3.022 ± 0.1196). Note the dotted black line indicates the mean value (*FCRL6* log2 = 3.47) of the 11 breast cancer subtypes.

We next investigated *FCRL6* expression among BRCA (*n* = 1,067) TCGA samples (Figure 4). *FCRL6* was able to predict progression free survival (PFS) among all BRCA samples with elevated transcript expression again correlating with a favorable outcome (Figure 4A). For *FCRL6*-high cases the median PFS was not reached for this TCGA BRCA cohort, while the median PFS for *FCRL6*-low cases was 113.8 months (HR = 0.44, CI = 0.22–0.86, *P* = 0.0025). Interestingly, analysis of OS indicated an advantage for patients with tumors possessing high *FCRL6* transcripts (Figure 4B). Median OS was 129.7 months for *FCRL6*-high cases and 107.2 months for patient samples with *FCRL6*-low expression (HR = 0.61, CI = 0.43–0.97, *P* = 0.0025). This benefit across this entire series of TCGA BRCA tumors appeared evident for up to 12 years after diagnosis, but for patients with *FCRL6*-high samples that lived beyond this time period (*n* = 10/19), this factor became detrimental. Notably, this is a minority of patients from a heterogeneous cohort of samples and mortality at later time points beyond diagnosis and treatment could be multifactorial for this group. With regard to the disease status of these 10 individuals, six were tumor-free at death, two died with positive tumor status, and data was not available for two cases. We additionally assessed TCGA BRCA samples from patients with Her2 negative status (*n* = 550) (Figures 4C,D). Elevated *FCRL6* expression in patients with Her2 negative tumors was prognostically advantageous for both PFS and OS. Median PFS for patients with *FCRL6* high Her2 negative tumors was not reached while for low expressors it was 113.8 months (HR = 0.44, CI = 0.22–0.86, *P* = 0.0025). Median OS for *FCRL6*-high Her2 negative BRCA tumors was also not reached and for *FCRL6*-low expression was 93.8 months (HR = 0.48, CI = 0.25–0.94, *P* = 0.0081). Elevated FCRL6 expression also portended significantly higher PFS and OS in patients with intraductal breast carcinoma (*n* = 765) and estrogen positive (*n* = 782) tumor status (data not shown). To better understand the distribution of *FCRL6*^+^ TILs within the context of BRCA heterogeneity, we analyzed the mean expression levels of *FCRL6* among 11 different BRCA subtypes. The highest *FCRL6* transcripts were found in TNBC, followed by ER^−^ > Basal > Her2^−^ tumor samples (Figure 5). These findings parallel the known elevated frequency of cytotoxic lymphocytes and NK cell TILs in TNBC that are associated with an improved prognosis as well as the sensitivity of these tumors to ICI and neoadjuvant chemotherapy (69, 72, 83–85). While analysis of FCRL6 protein expression by primary samples would be helpful for validation, given its restricted expression by cytotoxic lymphocytes, these findings at the transcript level support the potential utility of *FCRL6* as a prognostic marker and ICI target.

## Concluding Remarks

In summary, members of the FCRL family are preferentially expressed by B cells and generally exert inhibitory tyrosine-based regulation on BCR signaling. Given their expression by B cells there is a growing appreciation of their roles in lymphoproliferative disorders and potential as immunotherapeutic targets. In contrast to FCRL1–5, FCRL6 has a distinct expression pattern outside the B lineage among cytotoxic T and NK lymphocytes. Furthermore, its elevated expression in the tumor microenvironment, including NSCLC, melanoma, and breast cancer, significantly correlates with improved PFS and OS. However, its ITIM-based repressive function in these cells becomes operative upon engagement with its partner MHCII/HLA-DR. This newfound axis has growing significance in tumor immunology as endogenous HLA class II expression by cancer cells has been found to correlate with increased TIL numbers, responsiveness to anti PD-1 directed ICI, and a more favorable prognosis. However, some tumors that develop resistance to ICI appear to upregulate HLA class II to blunt recognition by cytotoxic cells expressing FCRL6 as well as other MHCII-binding molecules (e.g., LAG3 and NKp44). Thus, FCRL6 may serve as a novel ICI target. Future studies that model its *in vivo* regulation are required to investigate this possibility, but are hampered by the interspecies diversity of this FCRL representative in mice and humans. Additionally, the distinct MHCII allotypes that FCRL6 interacts with, and how these relationships impact cytotoxic cells during homeostasis in tolerance with APCs vs. disease states, are important topics for future study.

## Author Contributions

RSD wrote and edited the manuscript.

## Conflict of Interest

RSD has filed a patent on FCRL6-specific antibodies and their use in immunotherapy.
